# A Computational Cognitive Biomarker for Early-Stage Huntington’s Disease

**DOI:** 10.1371/journal.pone.0148409

**Published:** 2016-02-12

**Authors:** Thomas V. Wiecki, Chrystalina A. Antoniades, Alexander Stevenson, Christopher Kennard, Beth Borowsky, Gail Owen, Blair Leavitt, Raymund Roos, Alexandra Durr, Sarah J. Tabrizi, Michael J. Frank

**Affiliations:** 1 Cognitive, Linguistic & Psychological Sciences, Brown, Providence, United States of America; 2 Division of Clinical Neurology, Nuffield Department of Clinical Neurosciences, Level 6 West Wing, John Radcliffe Hospital, University of Oxford, Oxford, OX3 9DU, United Kingdom; 3 CHDI Management Inc/CHDI Foundation, 155 Village Boulevard, Suite 200, Princeton, NJ 08540, United States of America; 4 Huntington’s Disease Research Centre, UCL Institute of Neurology, 2nd Floor Russell Square House, 10-12 Russell Square, London, WC1B 5EH, United Kingdom; 5 Department of Medical Genetics, University of British Columbia, Vancouver, British Columbia V6T 2B5, Canada; 6 Department of Neurology, Leiden University Medical Centre, 2300RC Leiden, The Netherlands; 7 Department of Genetics and Cytogenetics, and INSERM UMR S679, APHP Hôpital de la Salpêtrière, 75013 Paris, France; Heidelberg University, GERMANY

## Abstract

Huntington’s disease (HD) is genetically determined but with variability in symptom onset, leading to uncertainty as to when pharmacological intervention should be initiated. Here we take a computational approach based on neurocognitive phenotyping, computational modeling, and classification, in an effort to provide quantitative predictors of HD before symptom onset. A large sample of subjects—consisting of both pre-manifest individuals carrying the HD mutation (pre-HD), and early symptomatic—as well as healthy controls performed the antisaccade conflict task, which requires executive control and response inhibition. While symptomatic HD subjects differed substantially from controls in behavioral measures [reaction time (RT) and error rates], there was no such clear behavioral differences in pre-HD. RT distributions and error rates were fit with an accumulator-based model which summarizes the computational processes involved and which are related to identified mechanisms in more detailed neural models of prefrontal cortex and basal ganglia. Classification based on fitted model parameters revealed a key parameter related to executive control differentiated pre-HD from controls, whereas the response inhibition parameter declined only after symptom onset. These findings demonstrate the utility of computational approaches for classification and prediction of brain disorders, and provide clues as to the underlying neural mechanisms.

## Introduction

Huntington’s disease (HD) is a debilitating neurodegenerative disease with progressive degradation of motor and cognitive function. From a neurocognitive perspective, HD is a highly interesting disorder as it has a clearly defined, single genetic mutation in the form of an expanded CAG repeat in the *HTT* gene, which predicts with certainty that the disease will develop in an individual. The effects of this mutation on neurobiology have been the subject of intense study with notable progress, although many questions still remain. Indeed, no clinical phase 3 trial to date has been successful for a drug that slows or reverses progression of HD, raising the question of whether the most efficient drug development methods are being leveraged [1]. A central requirement for success in clinical trials are objective and quantitative outcome measures that are sensitive to early-stage changes in presymptomatic individuals (pre-HD) as well as early stage manifest HD. Better clinical markers of disease progression could inform when to initiate treatment: too early would increase accumulation of negative side-effects, whereas too late could prevent successful therapeutic intervention.

TRACK-HD was a large multi-site longitudinal study to evaluate various behavioral and imaging measures for their appropriateness in tracking HD progression [2]. While many measures were sensitive to changes in early symptomatic HD, a key conclusion was that “these measures are insensitive to change in pre-HD over timescales realistic for clinical trials [3] and more sensitive measures are required to capture subtle changes that might be taking place before symptom onset.” [4]. In sum, there is a current lack of clinical markers sensitive to the cognitive changes that occur during the pre-HD stages.

Oculomotor abnormalities have been investigated, both at very early stages and even during the premanifest period by a number of studies [5–7]. The antisaccade conflict task has been widely used to study executive control and response inhibition of eye movements that has well-studied and dissociable neural mechanisms associated with (i) the prepotency of a pro-saccade response, (ii) the inhibition of that response, and (iii) the executive control needed to dictate the alternative response given the instructed task rule [8, 9]. Notably, several studies have found reliable antisaccade performance deficits in HD subjects well before full onset of HD symptoms [10–12].

Traditional studies with this task mostly analysed and interpreted behavioral summary statistics such as mean reaction time and accuracy. However, despite the apparent task simplicity, its successful completion involves an intricate interaction within a complex network of brain areas including the frontal cortex and basal ganglia. Indeed, neural circuit modeling and empirical studies suggest that a deficit in any of the involved areas can lead to increased error rates and reaction times, leading to ambiguity in interpretation of observed deficits [8]. The emerging field of computational psychiatry [13, 14] approaches this problem with the help of computational models that can deconstruct behavioral and neural data into separable generative processes, and to identify whether any of these processes is preferentially altered in mental illness [15].

At a mechanistic level, the classical view is that HD arises from selective neurodegeneration within the indirect pathway of the basal ganglia that normally acts to suppress unwanted movements [2, 16–20]. In addition to this clearly defined atrophy, there is also more widespread degeneration in frontal cortex [10, 11, 21], which could act to impair executive control over action selection [8, 22–24].

The aim of the current study was to apply quantitative computational modeling to the TRACK-HD behavioral data set (specifically, the antisaccade conflict task) to separate processes thought to relate to selective response inhibition and executive control. We then use machine learning classification to demonstrate that the executive control parameter is predictive of HD prior to symptom onset, whereas response inhibition processes are impaired only after motor symptoms are observed.

## Methods

This was a novel analysis of a multinational longitudinal observational study approved across sites as described in [2].

371 subjects performed an antisaccade task as part of the TRACK-HD study [2]. This task requires subjects to look away from an appearing stimulus on a screen. The data set consists of 123 healthy controls (mean age 46±10 years), 122 presymptomatic gene carriers (pre-HD; mean age 41±8.7 years) that will develop HD later in life, and 125 subjects diagnosed with HD (mean age 49.3±9.8 years). Pre-HD subjects were further subdivided into pre-HD-A and pre-HD-B, where pre-HD-B were estimated to be closer than pre-HD-A to progression to HD based on CAG repeat length and age [25]. Specifically, this group was split at the median predicted years to onset (10.8 years) into preHD-A (≤10.8 years from predicted onset) and preHD-B (<10.8 years). HD subjects were similarly divided using UHDRS total functional capacity (TFC) score. Participants with early HD were designated either HD-1 (TFC 11–13) or HD-2 (TFC 7–10), with HD-2 group having more advanced symptoms [26].

Several clinical measures were collected. The Unified Huntington’s Disease Rating Scale (UHDRS) is the standard assessment tool for HD symptom severity and has two relevant subscores: Total functional capacity (TFC), tracking ability to perform daily events, and the total motor score (TMS) tracking motor abilities specifically [27].

Mean and standard-deviaton (SD) of prosaccade RTs, mean and SD of correct and error antisaccade RTs, as well as accuracy on antisaccade trials were computed as summary statistics.

### Behavioral testing

Oculomotor testing was carried out using a “saccadometer advanced” (Ober consulting)—a head mounted oculometer using a miniaturized infra-red 1kHz camera to track eye movements [28]. Since it is head mounted, it sits comfortably on the patients’ nose and there is no need for head restraint. This methodology has been previously used successfully with Huntington’s patients [7].

**Fig 1:**
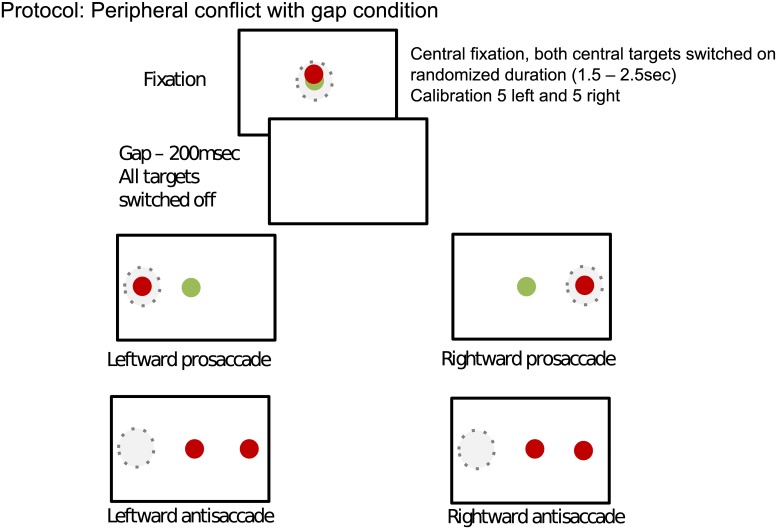
Structure of the antisaccade task. Participants first fixated on the center of the screen. Afterwards, a blank screen was presented followed by either a green or red central cue instructing participants to either make a pro or antisaccade, respectively.

The test starts with a pair of red and green lights presented in the center of a computer screen. The participants were directed to look at these lights to get ready. Next, the pair of lights turned off. Participants then would see the single colored light in the center and the red target off to the side. The participants were instructed that the color of the central cue would inform them to make a pro (i.e. green cue) or antisaccade (i.e. red cue) to or away from a target, respectively. Target location was randomly on the left or right side of the target (see Fig 1). Pro and antisaccades were randomly interleaved. Prosaccade errors were very rare (<1% of all trials) and not analyzed further.

### Distributional analysis

Summary statistics are a useful and easy measure to compute. But while mean and variance can describe a Gaussian distribution perfectly, RT distributions are well known to be quite skewed and non-normal. Thus, summary statistics often fail to capture more nuanced aspects of conflict resolution that are present in the full RT distributions of correct and error trials. Indeed, distributional analysis can help tease apart different processes that can lead to various changes in the RT distributions (due to conflict or other factors), such as a shift in the entire distribution, or preferential changes to the leading edge or the tails of the distribution, and how any such changes are related to increased or decreased accuracy [29–31]. Distributional analysis typically involves dividing the RT distribution into quantiles, e.g., the mean of the first 20% of the RT distribution, the second 20%, and so on.

In order to better capture differences in the RT distribution between congruent and incongruent trials, [29] suggested the use of delta-plots. For each subject, RT is split into 6 quantile ranges (0–10, 10–30, 30–50, 50–70, 70–90, 90–100) for pro and antisaccade trials separately (only correct antisaccade trials are used). Mean RTs of each range are then averaged across pro and antisaccade trials and plotted along the x-axis. To capture conflict-induced slowing, mean RT in each antisaccade range is subtracted from mean RT of the corresponding prosaccade range and plotted along the y-axis. Thus, the relative slowing for antisaccades compared to prosaccades is captured by a positive y-value in the delta plot. The commonly observed effect is that conflict effects are observed to a greater degree on early RTs, as captured by a decreasing slope of the delta-plot.

### Computational modeling

**Fig 2:**
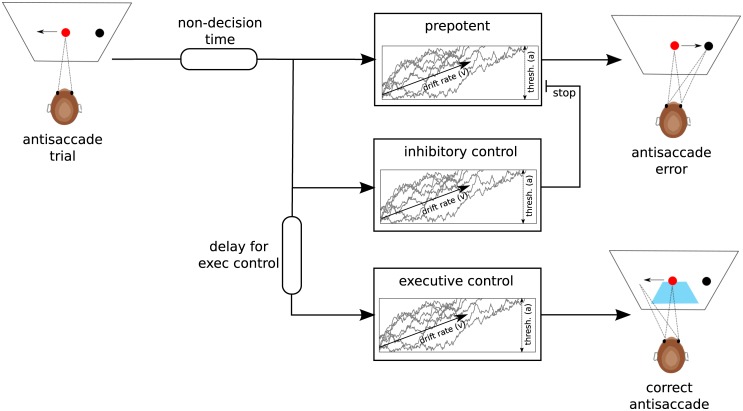
Computational process model of the antisaccade task. The architecture of accumulators during an antisaccade trial is depicted. During prosaccade trials, only the prepotent process is used. See the main text for a description of the model.

While the delta-plot can reveal behavioral signatures of conflict resolution it does not provide a process level description of how such signatures arise. To this end, we fit a computational model summarizing the three major components to the behavior in the task and which approximate those embedded in more detailed neural models. The model is an extension of a sequential sampling model typically used in two-alternative forced-choice decision making tasks, in which sensory evidence is accumulated up to a response threshold used to initiate motor activity, and where the speed of evidence accumulation is reflected by a “drift rate”. The extended model used here takes into account the dynamics and interactions of prepotent responses, response inhibition, and executive control. As such, the model comprises three single-boundary Wald accumulators: a prepotent (pre), an inhibitory (inhib) and an executive control (exec) accumulator (see Fig 2). These accumulators race against and interact with each other. Each accumulator is associated with an individual drift-rate (*v_pre_*, *v_inhib_* and *v_exec_*) that determines the speed of integration towards its threshold *a*. To take into account additional time unrelated to decision processes but summarizing sensory perception and motor execution, we also incorporate a non-decision time parameter *t*. If the prepotent accumulator reaches its threshold first during an antisaccade trial an error is commited. If the inhibitory accumulator reaches the threshold before the prepotent one, it stops the prepotent accumulator from reaching its threshold. In addition, the executive control accumulator is delayed by a fixed time (*t_exec_*) to capture additional time required for rule-retrieval, vector inversion etc. Once it reaches threshold a correct antisaccade is performed. While parameters of the prepotent accumulator (i.e. *v_pre_*, *a* and *t*) are identified by fitting across both pro and antisaccade trials, all other parameters are fit using only antisaccade trials (as they are irrelevant in prosaccade trials). This model was chosen from various configurations by performing model comparison (see the appendix).

As a closed-form solution to this likelihood is difficult to compute, we used probability density approximation (PDA) introduced by [32]. This likelihood-free method only requires simulation of data from a generative process and approximates a likelihood function using kernel density estimation. We can then easily evaluate the data on the approximated likelihood to compute the summed log probability and find the best fitting parameters using Powell optimization [33] with basin-hopping [34] to avoid getting stuck in local maxima. While ideally we would use hierarchical Bayesian estimation of the model parameters [35] the small randomness along with the large number of simulations required for a single evaluation of the PDA likelihood function lead to convergence issues and prohibitively long running times.

### Machine Learning

In order to assess the viability of using these methods to classify subjects, we used machine learning classifiers based on summary behavioral statistics and computational model parameters. The goal was to train classifiers based on behavioral and model parameters in a sample of subjects, and test whether the classifier could discriminate between novel groups of subjects. For two-class classification we used logistic regression with L2-regularization. To optimize the strength of the regularization parameter we ran 10-fold stratified cross-validation, which keeps the distribution of labels constant across every split. During cross-validation, the classifier is trained to differentiate 90% of the subjects but tested and evaluated based on its classification accuracy of the previously unseen 10% of subjects. This splitting procedure is repeated 10 times so that all data has been used once to test the classifier. To evaluate the performance of this classifier we ran this cross-validation procedure 200 times on training data and tested the best-performing classifier on held-out test data in a shuffle-split cross-validation with 20% of the data used for testing each time. Classifier performance was then compared using the Area Under the Receiver-Operator-Characteristic Curve (AUC), a measure robust to unequal class sizes. Intuitively, it can be interpreted as the probability of correctly classifying two samples randomly drawn from each of the classes. For multiclass classification we used a Random Forest classifier [36] that was trained in the same manner. We have experimented with various other more advanced ML classification algorithms including Support Vector Machines and Elastic Nets but had almost identical results and thus present the results using a simpler classifier.

## Results

### Behavior

**Fig 3:**
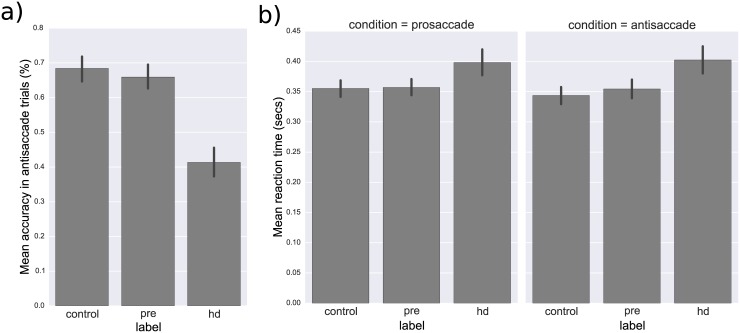
**a)** Bar-plots of mean reaction time (in seconds) across different groups. **b)** Bar-plots of mean percent accuracy during antisaccade trials across different groups. Error-bars depict 95% confidence intervals.

Standard measures of behavior were more than sufficient to discriminate HD subjects from both controls and pre-HD. Specifically, for prosaccade trials, control subjects t(246) = -3.25, p = 0.001) as well as pre-HD subjects (t(245) = -3.13, p = 0.002) were significantly faster (0.344±0.0806 secs and 0.357±0.0799 secs, respectively) than HD subjects (0.398±0.1226 secs; see Fig 3a). A similar pattern emerged in antisaccade trials where control subjects t(246) = -4.25, p < 0.001 as well as pre-HD subjects t(245) = -3.39, p = 0.001 were significantly faster (0.344±0.0806 secs and 0.355±0.0866 secs, respectively) than HD subjects (0.402±0.1308; see Fig 3a). Control subjects t(246) = 9.68, p < 0.001 as well as pre-HD subjects t(245) = 8.85, p < 0.001 were also more accurate (68.4±19.77% and 65.9±19.31%, respectively) than HD subjects (41.3±24.06%) on antisaccade trials.

Notably, there was no significant difference between control and pre-HD subjects in mean RT in either prosaccade t(243) = 0.15, p = 0.879 or antisaccade t(243) = 1.01, p = 0.315 trials, nor in antisaccade accuracy t(243) = -1.00, p = 0.318 (see Fig 3b). There was, however, a trend for pre-HD to demonstrate increase antisaccade RT variability (standard deviation) between pre-HD (0.139±0.0756 secs) and controls (0.122±0.0615 secs), t(243) = 1.95, p = 0.052.

### Distributional analysis

**Fig 4:**
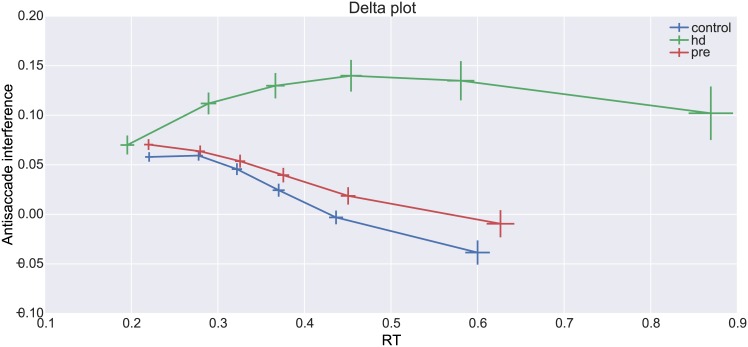
Delta-plot showing conflict resolution (negative slope) across time in different groups. Error-bars represent standard errors. See text for details.

Delta-plots subtract pro from antisaccade RTs for each quantile along the distribution and show the conflict interference effect (positive deflections) and how it gets resolved over time. The delta-plots for the three different subject groups are shown in Fig 4. The common pattern of a negative slope [37] is strongly visible in all groups and suggests that conflict is successfully resolved as time progresses. While there are striking differences in the last 3 quantiles between control and HD as well as pre-HD and HD (all p-values < 0.001) there were no differences between controls and pre-HD (all p-values >.05).

### Computational modeling

#### Separable effects of response inhibition and executive control

Before describing group differences, it is important to highlight that the model comprises multiple mechanisms by which a correct or incorrect antisaccade is executed. High values of *v_pre_* lead to faster prosaccades but also fast antisaccade errors. Both the response inhibition parameter *v_stop_*, which allows a prepotent saccade to be suppressed, and the executive control parameter *v_exec_*, which provides evidence for the controlled antisaccade response, contribute to successful performance (decreased errors). However, high values of *v_exec_* lead not only to higher accuracy but faster and less skewed correct antisaccade RTs. In contrast, high values of *v_stop_* do not affect antisaccade RTs but rather right-censor the antisaccade error RT distribution (i.e., erroneous pro-saccades will only occur with very fast RTs). Finally, longer *t_exec_* time will allow for more time for the prepotent process to reach threshold, and thus will also increase antisaccade errors, but does so by causing a constant shift forward of the whole RT distribution, accounting for the commonly observed pattern of relatively fast errors and delayed correct antisaccade RTs. Thus, each of the model parameters quantify separately identifiable cognitive processes (and putative underlying neural mechanisms). We verified through generative simulations and parameter recovery that indeed these parameters are separately identifiable.

### Model fit

**Fig 5:**
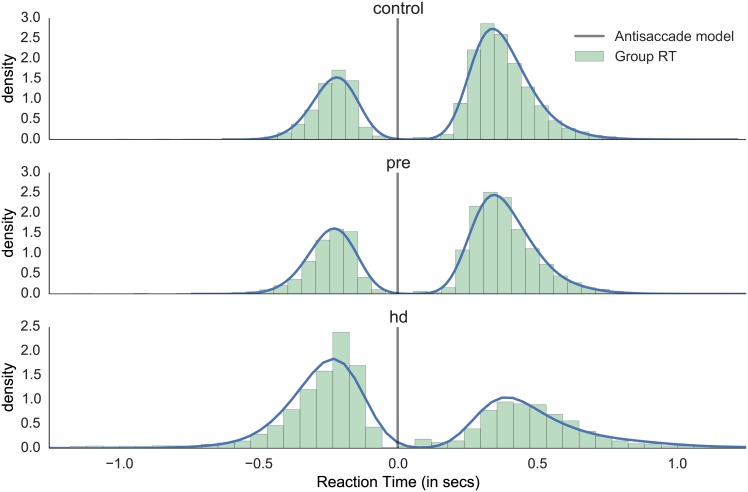
Model fit for antisaccade trials. Aggregate RT histograms were generated from the model with fitted parameters for each group, overlaid on top of empirical normalized aggregate RT histogram. Errors are mirrored along the y-axis and appear on the negative side.

Visual inspection of model fit to aggregate RT data shows that the model is able to capture the overall shape of correct and incorrect antisaccade trials across the three different groups (Fig 5). Note that the results reported elsewhere in this study rely on fits to individual subjects, the group fit is done for visualization purposes only (i.e. to assess how well the model captures the mean behavioral patterns).

For more analyses of model fit and model comparison we refer to the supplement.

#### Group differences

**Fig 6:**
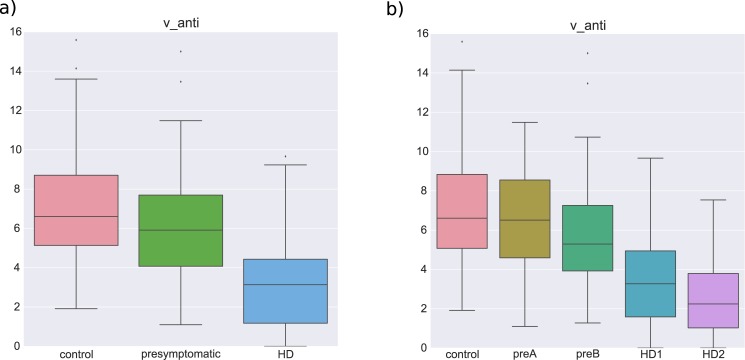
**a)** Box-plots of *v_exec_* in different groups. **b)** Box-plots of *v_exec_* in different subgroups.

Unsurprisingly, given the large behavioral differences between symptomatic HD subjects and both controls and pre-HD, all model parameters significantly differed between controls and HD as well as between pre-HD and HD (all p-values < 0.01). The more interesting question is whether the refined modeling could help to differentiate pre-HD from controls given that most traditional behavioral analyses revealed no clear differences. Notably, we found that the executive control drift-rate (*v_exec_*) was significantly lower t(243) = -2.66, p = 0.008 in pre-HD subjects (6.218±2.6506) compared to controls (7.101±2.5423; see Fig 6a). This finding suggests subtle executive control deficits in premanifest HD gene carriers. Moreover, visual analysis of changes in executive control drift-rate across subgroups of HD (Fig 6b) suggests a linear relationship between progression of HD and this parameter, as we assess next.

#### Correlations

A multiple linear regression between all model parameters and a linear coding of HD stage (controls = 0, pre-HD-A = 1, pre-HD-B = 2, HD-1 = 3, HD-2 = 4) revealed strong correlations between *v_exec_*, *t_exec_*, *v_inhib_* and HD stage. Overall, the model parameters explained 39% of the variance F(4,353) = 54.81, p < 0.001 (see Table 1).

**Table 1:** Results of multiple linear regression of model parameters on disease stage, where disease stage was coded linearly (controls = 0, pre-HD-A = 1, pre-HD-B = 2, HD-1 = 3, HD-2 = 4).

**Dep. Variable:**	stage	**R-squared:**	0.393
**Model:**	OLS	**Adj. R-squared:**	0.383
**Method:**	Least Squares	**F-statistic:**	38.08
**No. Observations:**	360	**AIC:**	1116.
**Df Residuals:**	353	**BIC:**	1143.

	coef	std err	t	P>|t|	[95.0% Conf. Int.]
**Intercept**	2.1898	0.298	7.357	0.000	1.604 2.775
***v_exec_***	-0.3031	0.027	-11.190	0.000	-0.356 -0.250
***v_pre_***	0.1298	0.068	1.905	0.058	-0.004 0.264
**a**	0.3380	0.124	2.726	0.007	0.094 0.582
**t**	-0.2199	1.271	-0.173	0.863	-2.719 2.279
***t_exec_***	2.7908	0.667	4.181	0.000	1.478 4.103
***v_inhib_***	-0.1274	0.029	-4.404	0.000	-0.184 -0.070

**Fig 7:**
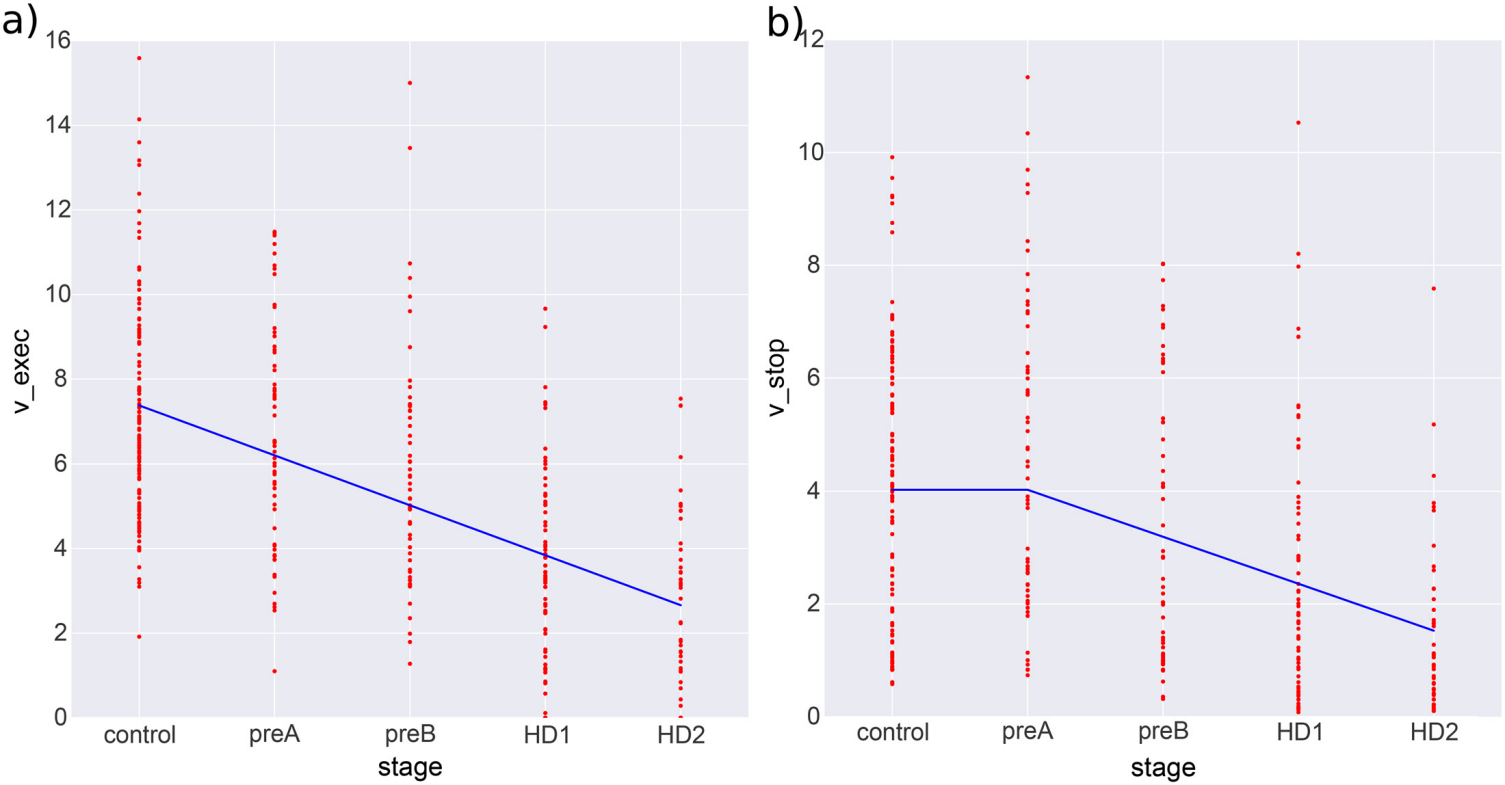
Multivariate Adaptive Regression Splines (MARS) estimation of a piece-wise linear relationship between *v_stop_* (a) and *v_exec_* (b). See text for more details.

While some parameters might show an impairment only after symptoms are evident (e.g., if the mechanisms of motor symptoms are related to the mechanisms producing the reduced model parameter), other parameters might show a more progressive signal even in the stages of pre-HD. We thus assessed for a piecewise linear relationship between parameters and disease stage using a Multivariate Adaptive Regression Spline (MARS) [38] regression. This iterative algorithm can detect break points in the linear relationship and model them explicitly. The results can be appreciated in Fig 7. While *v_exec_* shows a directly linear relationship, declining from early stages of pre-HD, *v_inhib_* seems to only change in later stages once motor symptoms are present. This fits with our group difference results that showed a significant difference between controls and pre-HD in *v_exec_*, but not in *v_inhib_*. Interestingly, these results suggest that executive control deficits occur *before* inhibitory control degradation that are only noticable after full HD onset.

**Fig 8:**
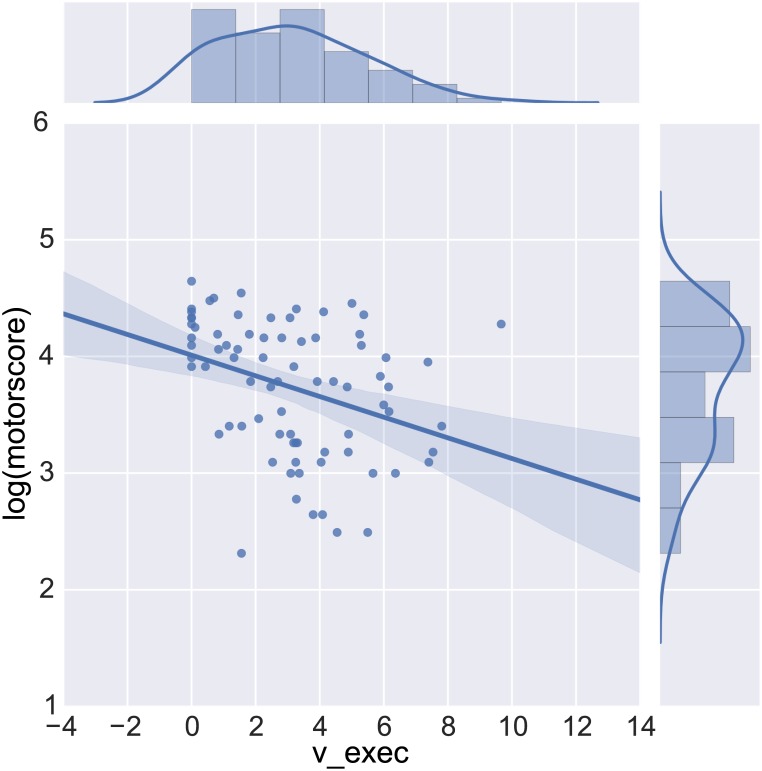
Best fitting linear regression line between *v_exec_* and log-transformed total motoro score (TMS) on top of raw subject scores.

Deficits in both *v_exec_* and *v_inhib_* were also strongly related to subjects’ TMS motor scores p < 0.001 (see Fig 8 and Table 2 for a multiple linear regression analysis). Moreover, model parameters were significantly correlated with TFC F(363, 6) = 19.74 and explained 24% of the variance (see Table 3 for details).

**Table 2:** Results of multiple linear regression of model parameters on total motor score (TMS)—higher scores indicate worse motor problems.

**Dep. Variable:**	TMS	**R-squared:**	0.399
**Model:**	OLS	**Adj. R-squared:**	0.389
**Method:**	Least Squares	**F-statistic:**	40.14
**No. Observations:**	370	**AIC:**	2714.
**Df Residuals:**	363	**BIC:**	2742.

	coef	std err	t	P>|t|	[95.0% Conf. Int.]
**Intercept**	14.7502	2.446	6.031	0.000	9.940 19.560
***v_exec_***	-2.4764	0.223	-11.109	0.000	-2.915 -2.038
***v_pre_***	1.0374	0.554	1.872	0.062	-0.052 2.127
**a**	3.0337	1.016	2.987	0.003	1.037 5.031
**t**	-4.3692	10.451	-0.418	0.676	-24.922 16.184
***t_exec_***	18.7905	5.513	3.409	0.001	7.950 29.631
***v_inhib_***	-1.2665	0.237	-5.334	0.000	-1.733 -0.800

**Table 3:** Results of multiple linear regression of model parameters on total functional capacity (TFC). Higher TFC indicates better functioning than lower scores.

**Dep. Variable:**	tfc	**R-squared:**	0.246
**Model:**	OLS	**Adj. R-squared:**	0.234
**Method:**	Least Squares	**F-statistic:**	19.74
**No. Observations:**	370	**AIC:**	1268.
**Df Residuals:**	363	**BIC:**	1296.

	coef	std err	t	P>|t|	[95.0% Conf. Int.]
**Intercept**	11.7465	0.347	33.890	0.000	11.065 12.428
***v_exec_***	0.2365	0.032	7.488	0.000	0.174 0.299
***v_pre_***	-0.0971	0.079	-1.236	0.217	-0.251 0.057
**a**	-0.3199	0.144	-2.223	0.027	-0.603 -0.037
**t**	0.3705	1.481	0.250	0.803	-2.542 3.283
***t_exec_***	-2.1401	0.781	-2.740	0.006	-3.676 -0.604
***v_inhib_***	0.1398	0.034	4.154	0.000	0.074 0.206

There was no correlation between any of the model parameters and the CAG repeat length in a multlinear regression R^2^ = 0.02, F(240, 6) = 0.8, p = 0.57.

### Machine Learning

**Fig 9:**
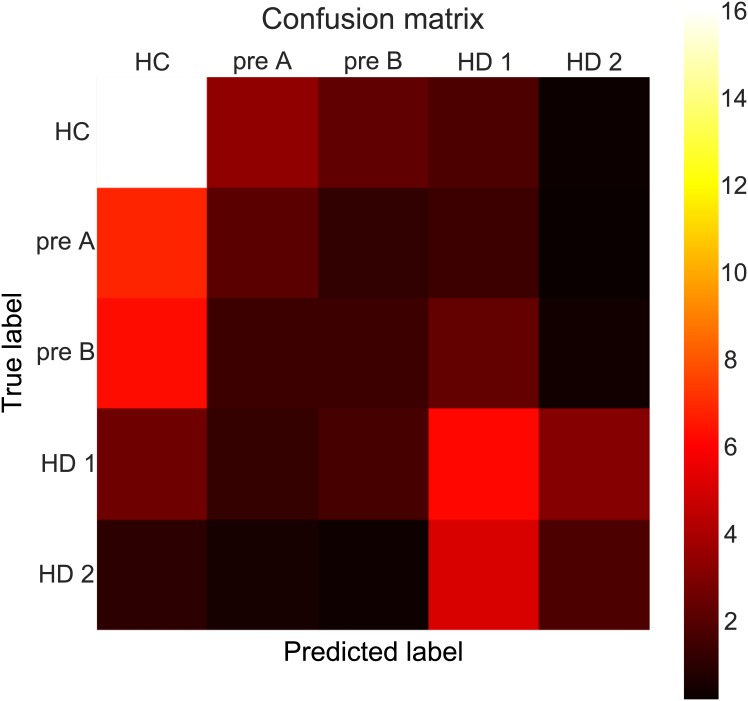
Confusion matrix showing true class labels as well as class labels predicted.

We next asked if disease state could be predicted using the model parameters alone. First, we wanted to assess how well each subgroup could be identified given only the model parameters. The confusion matrix in Fig 9 shows the results of training a random forest and testing its multiclass predictions on held-out data (i.e, predicting patient group status in subjects for whom the training procedure had not seen). The classifier achieves an accuracy of 40% which is modestly above chance (i.e. 33% due to class imbalances).

**Fig 10:**
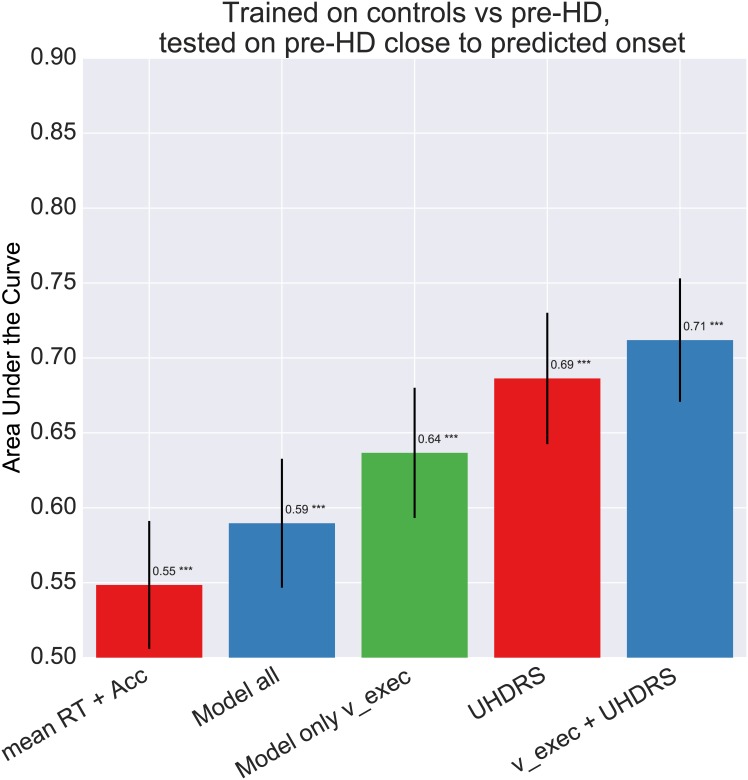
Bar-plot comparing Area under the ROC Curve (AUC) of a logistic regression classifier trained to differentiate pre-HD subjects from controls, evaluated specifically here on its performance predicting pre-HD-B. Error-bars represent standard deviation.

Next we evaluated how well the above classifer was able to discriminate pre-HD-B subjects—those presymptomatic patients closer to converting—from controls. As can be seen in Fig 10, the success of the classifer was improved when considering pre-HD-B subjects, particularily when the classifiers were trained on both model parameters and UHDRS scores. Summary statistics were not sensitive to this pattern and had significantly lower AUC than all other classifiers (all p-values < 0.0001). Interestingly, combining *v_exec_* with UHDRS scores also leads to higher accuracy than using UHDRS alone.

The next clinical setting we consider is whether a classifier can discriminate between controls and non-symptomatic subjects carrying the CAG repeat mutation. This application could be of interest if any signal picked up by the classifier could help identify pre-HD subjects that might be closer to converting to symptom onset. We compare classifier performance when trained on behavioral summary data (mean and SD RT in pro and antisaccade trials as well as accuracy in antisaccade trials), versus when it is trained on the discriminative model parametes *v_exec_*, versus when it is trained on the standard UHDRS assessment score consisting of TMS and TFC. The AUC of the classifiers on held-out data can be appreciated in Figure A in S1 File. All classifiers were significantly better than chance (all p-values < 0.05). As can be seen, UHDRS provides the highest level accuracy (p < 0.001) followed by *v_exec_*, followed by all model parameters, and finally the summary scores (p < 0.001) which operate close to chance.

In the case where we know if a patient has the CAG repeat mutation it is relevant to classify how close a pre-HD individual is to progressing to manifest HD. We thus trained a classifier to predict subgroups pre-HD-A and pre-HD-B. As can be seen in Figure B in S1 File, our previously identified parameter *v_exec_* results in the highest accuracy. However, significance testing only relevealed a trend (p = 0.089) when comparing *v_exec_* to UHDRS scores and no significant difference when comparing accuracy using all model parameters to UHDRS (p = 0.28). All parameters, *v_exec_* and UHDRS significantly outperformed RT summary measures (all p-values < 0.001). All classifiers were significantly different from chance (all p-values < 0.001). While the combination of *v_exec_* and UHDRS scores suggest a slight improvement, this difference was not significant (p = 0.12).

## Discussion

We demonstrated that computational methods based on the antisaccade behavioral data are useful in detecting subtle differences between non-symptomatic HD subjects and controls, and between different stages of pre-HD. As in earlier reports, manifest HD subjects had longer, more variable RTs as well as increased error rates in antisaccade trials [10–12]. This result was echoed by our analysis using delta-plots. We then fit a computational model inspired by [30] that decomposes the behavior on the antisaccade task into cognitive processes that quantify prepotent response tendencies, speed of inhibitory control to stop the prepotent response when it is maladaptive, and speed and onset time of executive control to initiate volitional saccades. The HD group was associated with differences in every model parameter, suggesting wide-spread neurodegeneration in this group. In contrast, the pre-HD group was selectively associated with deficits in executive control parameter, accompanied by skewed correct antisaccade trials

The pre-HD stage has mostly been in association with response inhibition deficits assumed to result from indirect pathway degeneration [2, 16–20, 39]. The indirect pathway of the BG has been suggested to provide a selective NoGo signal that suppresses maladaptive response tendencies [40–42]. Only in later stages, once motor-symptoms set in, other areas become impacted, such as other BG nuclei (subthalamic nucleus and substantia nigra), the thalamus, as well as cerebellum, cortex, and brainstem [43–45]. Contrary to this theory, our modeling results suggest that the early deficits observed in selective response inhibition tasks such as the antisaccade task result from executive control deficits rather than reduced response inhibition per-se. This result could suggest that it might not be indirect pathway degeneration that occurs in the early, pre-HD stages but rather frontal or fronto-striatal degradation. Our elaborated neural model of these tasks identify a pathway from prefrontal cortex to striatum that is involved in executive control to facilitate an adaptive rule-based response [8]. This theory is corroborated by a diffusion tensor imaging study that found reductions in white matter fibers projecting from the FEF to the caudate body of the BG in pre-HD individuals [10]. The amount of this degradation, as well as the UHDRS motor score [11], are associated with increased RT variability in voluntarily guided saccades, consistent with our findings and with a reduction in drift-rate [31, 46]. Furthermore, some evidence suggests that pre-HD is actually associated with *increased* indirect pathway activity [47], perhaps needed to counteract prepotent response tendencies when executive control is weakened. A recent study [21] also suggests that deficits in inhibitory control tasks like the stop-signal task are related to reduced activation of frontal areas such as the pre-supplementary motor cortex (pre-SMA) and dorsal anterior cingulate cortex (dACC). Note, however, that this theory rests on a link between the executive drift-rate parameter and frontal function which has theoretical linking in neural simulations [8], but has not been firmly established. Nevertheless, some recent data provides some supporting evidence from fMRI that the SMA is related to alterations in drift rate during executive control [48].

A second explanation of our finding is that it is indeed caused by indirect pathway degradation but in parts of the BG responsible for executive control which could in principle be affected in earlier disease stages than parts of the BG responsible for motor control. The BG has traditionally been associated with gating motor commands [49]. However, more recently it was shown that it also is involved in higher cognitive processing such as working memory updating [50–53]. Anatomically, the BG is known to form loops that originate in cortex, innervate the BG, and connect back up to the cortex via the thalamus in highly structured circuits [54]. Dorso-lateral PFC (DLPFC) is associated with executive control [22, 55] and consistently activated in antisaccade trials [56–58]. Notably, DLPFC innervates anatomical regions of the BG distinct from certain motor areas relevant for saccade generation (including FEF [59], SEF [60] and pre-SMA [61–63]). This alternative account thus suggests that indirect pathway degradations first happen in the BG areas innervated by DLPFC and only later progresses to areas innervated by motor cortex. However at this time, no clear mechanism is known which would lead to this progression within the BG.

These results might also be relevant for clinical and pharmaceutical research. Currently, there are no clinically proven therapies that could reverse the cognitive decline associated with the symptomatic stages of this disease. Thus, as with other neuronal disorders like Alzheimer’s disease, focus in the clinic has shifted towards early intervention to slow the progression which requires detection of subtle cognitive changes before the symptoms become visible neurologically.

Unfortunately, neither summary statistics nor delta-plots showed significant differences between control subjects and pre-HD individuals. Strikingly, however, our computational modeling analysis did show a significant difference in the drift-rate parameter for executive control (*v_exec_*). Moreover, when splitting subjects into subgroups a linear relationship between *v_exec_* and the progressive stages from early pre-HD to late HD emerged. Other model parameters associated with inhibitory control *v_inhib_*, delay of executive control, prepotent response bias, response caution and motor execution were only affected in HD subjects suggesting non-linear degradation of the various cognitive processes involved in the antisaccade task.

The computational approach provided several advantages. The model allowed us to detect an effect between controls and pre-HD. Moreover, the affected parameter allows for a more cognitive interpretation of the results. Our classification results show that the model parameters, specifically the above identified *v_exec_* parameter can provide higher classification accuracy than RT summary statistics, albeit not by a huge margin. The accuracies overall were not higher than the current clinical standard UHDRS. Moreover, the classifier was more successful in specifically discriminating pre-HD-B subjects from controls, suggesting that it could potentially detect subjects that are closer to reaching motor symptom onset. This hope awaits further data after more subjects have converted to be tested. Moreover, in a clinical setting we would likely use a battery of various cognitive tasks that could increase classification accuracy. The fact that data from a single task is competitive with UHDRS in certain circumstances is thus encouraging.

Ultimately, the hope is to identify measures that are more sensitive than TFC and TMS which are of limited clinical use to track disease progression in pre-HD [3]. As *v_exec_* showed correlations with these measures it could be such a clinical marker but it would require more validation and further analysis on longitudinal data to establish it as such.

## Limitations

A limiting factor of our computational analysis is that it assesses model parameters that guide inhibitory control without regard for sequential effects. A large body of literature shows that such effects do impact performance in conflict and inhibition tasks, such that conflict, errors or inhibitory demands on the previous trial all influence performance on the next trial [64–67]. However, our computational model is already somewhat complex, in that it contains multiple free parameters, and investigating how sequential effects further modulate these parameters poses an additional challenge for quantitative fitting, given that these parameters are somewhat collinear with each other. Future research should develop hierarchical Bayesian parameter estimation methods [35] which improve the ability to infer model parameters and to deal with such collinearity, but such methods benefit strongly from a closed form analytic solution to the likelihood of observations given the model. This solution is available for simpler sequential sampling models like the drift diffusion model used in other studies, but the model we used here to simulate the temporally evolving dynamics of inhibitory control does not benefit from that luxury. Addressing such limitations to assess the degree to which trial-to-trial behavioral adjustments are altered as a function of disease stage may be able to further improve classification performance.

## Supporting Information

S1 File Supporting information. Algorithm A, Algorithm to draw a single antisaccade trial. InvGaussian(*μ*, *λ*) is the first-passage-time distribution for a Wiener diffusion process with drift *μ* and a single upper threshold *λ*. *t* is a constant corresponding to non-decision time. *t*_*exec*_ is a second constant that captures the time needed to implement additional processing on antisaccade versus prosaccade trials. By running this algorithm 10000 times for each parameter setting and using kernel density estimation on the simulated RTs, we approximated a likelihood function. Prosaccade trials were fit using only the prepotent accumulator for which a closed-form solution is available. The parameters *a*, *t* and *v*_*pre*_ are thus constrained by prosaccade as well as antisaccade trials while the parameters *v*_*stop*_, *v*_*exec*_, and *t*_*exec*_ are only constrained by the latter. Figure A, Bar-plots of behavioral mean reaction time in seconds across different groups in the antisaccade condition. Ellipses in grey represent mean RT of trials simulated from models fit to each individual subject. Height of the ellipses represents standard-deviation. Figure B, Bar-plot comparing Area under the ROC Curve (AUC) of a logistic regression classifier trained on different data to predict HC and pre-HD. Error-bars represent standard deviation. Figure C, Bar-plot comparing Area under the ROC Curve (AUC) of a logistic regression classifier trained on different data to predict pre-HD-A and pre-HD-B. Error-bars represent standard deviation. Table A, AIC values of different model configurations. Lower AIC values represent a better trade-off between parsimony and model fit. Table B, Multiple Comparison of Means of *t* parameter—Tukey HSD. Table C, Multiple Comparison of Means of *v*_*pro*_ parameter—Tukey HSD. Table D, Multiple Comparison of Means of *v*_*stop*_ parameter—Tukey HSD. Table E, Multiple Comparison of Means of *v*_*exec*_ parameter—Tukey HSD. Table F, Multiple Comparison of Means of *t*_*exec*_ parameter—Tukey HSD. Table G, Multiple Comparison of Means of *a*_*mean*_ parameter—Tukey HSD. Table H, Multiple Comparison of Means of *a*_*diff*_ parameter—Tukey HSD.
(PDF)

## References

[1] Kieburtz K, Venuto C. TRACK-HD: both promise and disappointment. Lancet neurology. 2012 1;11(1):24–5. Available from: http://www.ncbi.nlm.nih.gov/pubmed/22137355. 10.1016/S1474-4422(11)70285-X 22137355

[2] Tabrizi SJ, Langbehn DR, Leavitt BR, Roos RA, Durr A, Craufurd D, et al Biological and clinical manifestations of Huntington’s disease in the longitudinal TRACK-HD study: cross-sectional analysis of baseline data. Lancet neurology. 2009 9;8(9):791–801. Available from: http://www.sciencedirect.com/science/article/pii/S147444220970170X. 10.1016/S1474-4422(09)70170-X 19646924 PMC3725974

[3] Tabrizi SJ, Scahill RI, Owen G, Durr A, Leavitt BR, Roos Ra, et al Predictors of phenotypic progression and disease onset in premanifest and early-stage Huntington’s disease in the TRACK-HD study: analysis of 36-month observational data. Lancet neurology. 2013 7;12(7):637–49. Available from: http://www.ncbi.nlm.nih.gov/pubmed/23664844. 10.1016/S1474-4422(13)70088-7 23664844

[4] Andre R, Scahill RI, Haider S, Tabrizi SJ. Biomarker development for Huntington’s disease. Drug discovery today. 2014 3;00(00):2–9. Available from: http://www.ncbi.nlm.nih.gov/pubmed/24632006. 10.1016/j.drudis.2014.03.002 24632006

[5] Golding CVP, Danchaivijitr C Hodgson TL Tabrizi SJ Kennard C. Identification of an oculomotor biomarker of preclinical Huntington disease. Neurology. 2006 8;67(3):485–7. Available from: http://www.neurology.org/content/67/3/485.short. 16625001 10.1212/01.wnl.0000218215.43328.88

[6] Blekher T, Weaver MR, Cai X, Hui S, Marshall J, Jackson JG, et al Test-retest reliability of saccadic measures in subjects at risk for Huntington disease. Investigative ophthalmology & visual science. 2009 12;50(12):5707–11. Available from: http://iovs.arvojournals.org/article.aspx?articleid=2185144. 10.1167/iovs.09-3538 19553607

[7] Antoniades CA, Altham PME, Mason SL Barker RA Carpenter R. Saccadometry: a new tool for evaluating presymptomatic Huntington patients. Neuroreport. 2007 7;18(11):1133–6. Available from: http://journals.lww.com/neuroreport/Abstract/2007/07160/Saccadometry__a_new_tool_for_evaluating.9.aspx http://www.ncbi.nlm.nih.gov/pubmed/17589313. 10.1097/WNR.0b013e32821c560d 17589313

[8] Wiecki TV, Frank MJ.A computational model of inhibitory control in frontal cortex and basal ganglia. Psychological Review. 2013 4;120(2):329–355. Available from: http://www.ncbi.nlm.nih.gov/pubmed/23586447. 10.1037/a0031542 23586447

[9] Watanabe M, Munoz DP. Probing basal ganglia functions by saccade eye movements. The European Journal of Neuroscience. 2011 6;33(11):2070–2090. Available from: http://www.ncbi.nlm.nih.gov/pubmed/21645102. 10.1111/j.1460-9568.2011.07691.x 21645102

[10] Klöppel S, Draganski B, Golding CV, Chu C, Nagy Z, Cook Pa, et al White matter connections reflect changes in voluntary-guided saccades in pre-symptomatic Huntington’s disease. Brain: a journal of neurology. 2008 1;131(Pt 1):196–204. Available from: http://www.ncbi.nlm.nih.gov/pubmed/18056161. 18056161 10.1093/brain/awm275

[11] Peltsch a, Hoffman a, Armstrong I, Pari G, Munoz DP. Saccadic impairments in Huntington’s disease. Experimental brain research. 2008 4;186(3):457–69. Available from: http://www.ncbi.nlm.nih.gov/pubmed/18185924. 10.1007/s00221-007-1248-x 18185924

[12] Hicks SL, Robert MPa, Golding CVP, Tabrizi SJ Kennard C. Oculomotor deficits indicate the progression of Huntington’s disease. Progress in brain research. 2008 1;171(08):555–8. Available from: http://www.ncbi.nlm.nih.gov/pubmed/18718352. 18718352 10.1016/S0079-6123(08)00678-X

[13] Montague PR, Dolan RJRRJ, Friston KJKKJ, Dayan P. Computational psychiatry. Trends in Cognitive Sciences. 2011 12;16(1):1–9. Available from: http://www.pubmedcentral.nih.gov/articlerender.fcgi?artid=3556822&tool=pmcentrez&rendertype=abstract http://linkinghub.elsevier.com/retrieve/pii/S1364661311002518 http://www.sciencedirect.com/science/article/pii/S1364661311002518. 22177032 10.1016/j.tics.2011.11.018 PMC3556822

[14] Maia TV, Frank MJ. From reinforcement learning models to psychiatric and neurological disorders. Nature neuroscience. 2011 2;14(2):154–62. Available from: http://www.ncbi.nlm.nih.gov/pubmed/21270784. 10.1038/nn.2723 21270784 PMC4408000

[15] Wiecki TV, Poland J, Frank MJ. Model-Based Cognitive Neuroscience Approaches to Computational Psychiatry: Clustering and Classification. Clinical Psychological Science. 2015 3;3(3):378–399. Available from: http://cpx.sagepub.com/content/3/3/378.full. 10.1177/2167702614565359

[16] Aylward EH, Sparks BF, Field KM, Yallapragada V, Shpritz BD, Rosenblatt A, et al Onset and rate of striatal atrophy in preclinical Huntington disease. Neurology. 2004 7;63(1):66–72. Available from: http://www.ncbi.nlm.nih.gov/pubmed/15249612. 10.1212/01.WNL.0000132965.14653.D1 15249612

[17] Hobbs NZ, Henley SMD, Wild EJ Leung KK Frost C Barker RA et al Automated quantification of caudate atrophy by local registration of serial MRI: evaluation and application in Huntington’s disease. NeuroImage. 2009 10;47(4):1659–65. Available from: http://www.ncbi.nlm.nih.gov/pubmed/19523522. 10.1016/j.neuroimage.2009.06.003 19523522

[18] Paulsen JS, Nopoulos PC, Aylward E, Ross CA, Johnson H, Magnotta VA, et al Striatal and white matter predictors of estimated diagnosis for Huntington disease. Brain research bulletin. 2010 5;82(3–4):201–7. Available from: http://www.sciencedirect.com/science/article/pii/S0361923010000705. 20385209 10.1016/j.brainresbull.2010.04.003 PMC2892238

[19] Majid DSA, Stoffers D Sheldon S Hamza S Thompson WK Goldstein J et al Automated structural imaging analysis detects premanifest Huntington’s disease neurodegeneration within 1 year. Movement disorders: official journal of the Movement Disorder Society. 2011 7;26(8):1481–8. Available from: http://www.pubmedcentral.nih.gov/articlerender.fcgi?artid=3136652&tool=pmcentrez&rendertype=abstract. 10.1002/mds.23656 21484871 PMC3136652

[20] Majid DSA, Aron AR Thompson W Sheldon S Hamza S Stoffers D et al Basal ganglia atrophy in prodromal Huntington’s disease is detectable over one year using automated segmentation. Movement disorders: official journal of the Movement Disorder Society. 2011 12;26(14):2544–51. Available from: http://www.ncbi.nlm.nih.gov/pubmed/21932302. 10.1002/mds.23912 21932302 PMC5615846

[21] Rao JA, Harrington DL, Durgerian S, Reece C, Mourany L, Koenig K, et al Disruption of response inhibition circuits in prodromal Huntington disease. Cortex. 2014 9;58:72–85. Available from: http://www.ncbi.nlm.nih.gov/pubmed/24959703. 10.1016/j.cortex.2014.04.018 24959703 PMC4227536

[22] Miller EK, Cohen JD. An integrative theory of prefrontal cortex function. Annual Review of Neuroscience. 2001;24:167–202. Available from: http://www.ncbi.nlm.nih.gov/pubmed/11283309. 10.1146/annurev.neuro.24.1.167 11283309

[23] Badre D. Cognitive control, hierarchy, and the rostro-caudal organization of the frontal lobes. Trends in cognitive sciences. 2008 5;12 Available from: http://www.ncbi.nlm.nih.gov/pubmed/18403252. 10.1016/j.tics.2008.02.004 18403252

[24] Collins AGE, Frank MJ. Cognitive control over learning: Creating, clustering, and generalizing task-set structure. Psychological Review. 2013 1;120(1):190–229. Available from: http://www.ncbi.nlm.nih.gov/pubmed/23356780. 10.1037/a0030852 23356780 PMC3974273

[25] Langbehn DR, Brinkman RR, Falush D, Paulsen JS, Hayden M. A new model for prediction of the age of onset and penetrance for Huntington’s disease based on CAG length. Clinical genetics. 2004;65(4):267–277. 10.1111/j.1399-0004.2004.00241.x 15025718

[26] Shoulson I, Fahn S. Huntington disease clinical care and evaluation. Neurology. 1979;29(1):1–1. 154626 10.1212/wnl.29.1.1

[27] Klempír J, Klempírova O, Spacková N, Zidovská J, Roth J. Unified Huntington’s disease rating scale: clinical practice and a critical approach. Functional neurology. 2005;21(4):217–21. Available from: http://www.ncbi.nlm.nih.gov/pubmed/17367582 http://europepmc.org/abstract/MED/17367582. 17367582

[28] Ober J, Przedpelska-Ober E, Gryncewicz W, Dylak J, Carpenter R, Ober J. Hand-held system for ambulatory measurement of saccadic durations of neurological patients. Modellingand Measurement in Medicine PAN, Warsaw. 2003;p. 187–198.

[29] Ridderinkhof KR, Scheres A, Oosterlaan J, Sergeant Ja. Delta plots in the study of individual differences: new tools reveal response inhibition deficits in AD/Hd that are eliminated by methylphenidate treatment. Journal of abnormal psychology. 2005 5;114(2):197–215. Available from: http://www.ncbi.nlm.nih.gov/pubmed/15869351. 10.1037/0021-843X.114.2.197 15869351

[30] Noorani I, Carpenter RHS. Antisaccades as decisions: LATER model predicts latency distributions and error responses. The European journal of neuroscience. 2012 11;37(September):1–9. Available from: http://www.ncbi.nlm.nih.gov/pubmed/23121177. 23121177 10.1111/ejn.12025

[31] Ratcliff R, McKoon G. The diffusion decision model: theory and data for two-choice decision tasks. Neural computation. 2008 4;20:873–922. Available from: http://www.ncbi.nlm.nih.gov/pubmed/18085991. 10.1162/neco.2008.12-06-420 18085991 PMC2474742

[32] Turner BM, Sederberg PB. A generalized, likelihood-free method for posterior estimation. Psychonomic bulletin & review. 2013 11;Available from: http://www.ncbi.nlm.nih.gov/pubmed/24258272. 10.3758/s13423-013-0530-0 PMC4143986 24258272

[33] Powell MJD. An efficient method for finding the minimum of a function of several variables without calculating derivatives. The Computer Journal. 1964 2;7(2):155–162. Available from: http://comjnl.oxfordjournals.org/content/7/2/155.abstract. 10.1093/comjnl/7.2.155

[34] Wales DJ, Doye JPK. Global Optimization by Basin-Hopping and the Lowest Energy Structures of Lennard-Jones Clusters Containing up to 110 Atoms. The Journal of Physical Chemistry A. 1997 7;101(28):5111–5116. Available from: 10.1021/jp970984n. 10.1021/jp970984n

[35] Wiecki TV, Sofer I, Frank MJ. HDDM: Hierarchical Bayesian estimation of the Drift-Diffusion Model in Python. Frontiers in neuroinformatics. 2013;7 Available from: http://www.ncbi.nlm.nih.gov/pubmed/23935581. 10.3389/fninf.2013.00014 PMC3731670 23935581

[36] Breiman L. Random forests.Machine learning. 2001;p. 1–33. Available from: http://link.springer.com/article/10.1023/A:1010933404324.

[37] Richard Ridderinkhof K Forstmann BU Wylie Sa Burle B van den Wildenberg WPM. Neurocognitive mechanisms of action control: resisting the call of the Sirens. Wiley Interdisciplinary Reviews: Cognitive Science. 2011 3;2(2):174–192. Available from: http://doi.wiley.com/10.1002/wcs.99. 26302009 10.1002/wcs.99

[38] Friedman JH. Multivariate Adaptive Regression Splines. The Annals of Statistics. 1991 3;19(1):1–67. Available from: http://projecteuclid.org/euclid.aos/1176347963. 10.1214/aos/1176347973

[39] Majid DSA, Cai W Corey-Bloom J Aron AR. Proactive selective response suppression is implemented via the basal ganglia. The Journal of neuroscience: the official journal of the Society for Neuroscience. 2013 8;33(33):13259–69. Available from: http://www.ncbi.nlm.nih.gov/pubmed/23946385. 10.1523/JNEUROSCI.5651-12.2013 23946385 PMC3742918

[40] Frank MJ. Dynamic dopamine modulation in the basal ganglia: A neurocomputational account of cognitive deficits in medicated and non-medicated Parkinsonism. Journal of Cognitive Neuroscience. 2005 1;17:51–72. 10.1162/0898929052880093 15701239

[41] Collins AG, Frank MJ. Opponent actor learning (OpAL): Modeling interactive effects of striatal dopamine on reinforcement learning and choice incentive. Psychological review. 2014;121(3):337 10.1037/a0037015 25090423

[42] Kravitz AV, Tye LD, Kreitzer AC. Distinct roles for direct and indirect pathway striatal neurons in reinforcement. Nature neuroscience. 2012 4;Available from: http://www.ncbi.nlm.nih.gov/pubmed/22544310. 10.1038/nn.3100 PMC3410042 22544310

[43] Johnson K, Cunnington R, Iansek R, Bradshaw J, Georgiou N, Chiu E. Movement-related potentials in Huntington’s disease: movement preparation and execution. Experimental Brain Research. 2001 6;138(4):492–499. Available from: http://link.springer.com/10.1007/s002210100733. 10.1007/s002210100733 11465748

[44] Kassubek J, Juengling FD, Ecker D, Landwehrmeyer GB. Thalamic atrophy in Huntington’s disease co-varies with cognitive performance: a morphometric MRI analysis. Cerebral cortex (New York, NY: 1991). 2005 6;15(6):846–53. Available from: http://www.ncbi.nlm.nih.gov/pubmed/15459079. 10.1093/cercor/bhh185 15459079

[45] MacMillan J, Quarrell O. The neurobiology of Huntington’s disease. MAJOR PROBLEMS IN NEUROLOGY. 1996;31:317–358.

[46] Wagenmakers EJ, Maas HLJ, Grasman RPPP. An EZ-diffusion model for response time and accuracy. Psychonomic Bulletin & Review. 2007 2;14(1):3–22. Available from: http://www.springerlink.com/index/10.3758/BF03194023. 10.3758/BF03194023 17546727

[47] Milnerwood AJ, Gladding CM, Pouladi MA, Kaufman AM, Hines RM, Boyd JD, et al Early increase in extrasynaptic NMDA receptor signaling and expression contributes to phenotype onset in Huntington’s disease mice. Neuron. 2010 1;65(2):178–90. Available from: http://www.sciencedirect.com/science/article/pii/S0896627310000139. 10.1016/j.neuron.2010.01.008 20152125

[48] Dunovan K, Lynch B, Molesworth T Verstynen T. Competing basal-ganglia pathways determine the difference between stopping and deciding not to go. eLife. 2015;p. e08723. 26402462 10.7554/eLife.08723 PMC4686424

[49] Mink JW. The Basal Ganglia: Focused selection and inhibition of competing motor programs. Progress in Neurobiology. 1996 03;50:381–425. Available from: http://www.ncbi.nlm.nih.gov/pubmed/9004351. 10.1016/S0301-0082(96)00042-1 9004351

[50] Frank MJ, Loughry B, O’Reilly RC. Interactions between the frontal cortex and basal ganglia in working memory: A computational model. Cognitive, Affective, and Behavioral Neuroscience. 2001 1;1:137–160. 10.3758/CABN.1.2.137 12467110

[51] McNab F, Klingberg T. Prefrontal cortex and basal ganglia control access to working memory. Nature Neuroscience. 2008 12;11(1):103–107. Available from: http://www.ncbi.nlm.nih.gov/pubmed/18066057. 10.1038/nn2024 18066057

[52] Baier B, Karnath HO, Dieterich M, Birklein F, Heinze C, Muller NG. Keeping Memory Clear and Stable–The Contribution of Human Basal Ganglia and Prefrontal Cortex to Working Memory. J. 2010 7;30(29):9788–9792. Available from: http://www.jneurosci.org/cgi/content/abstract/30/29/9788. 10.1523/JNEUROSCI.1513-10.2010 PMC6632833 20660261

[53] Chatham C, Frank M, Badre D. Corticostriatal output gating during selection from working memory. Neuron. 2014 1;81(4):930–942. 10.1016/j.neuron.2014.01.002 24559680 PMC3955887

[54] Alexander GE, DeLong MR, Strick PL. Parallel organization of functionally segregated circuits linking basal ganglia and cortex. Annual Review of Neuroscience. 1986 05;9:357–381. Available from: http://www.ncbi.nlm.nih.gov/pubmed/3085570. 10.1146/annurev.ne.09.030186.002041 3085570

[55] Chambers CD, Garavan H, Bellgrove MA. Insights into the neural basis of response inhibition from cognitive and clinical neuroscience. Neuroscience & Biobehavioral Reviews. 2009 5;33(5):631–646. Available from: 10.1016/j.neubiorev.2008.08.016. 10.1016/j.neubiorev.2008.08.016 18835296

[56] Wegener SP, Johnston K, Everling S. Microstimulation of monkey dorsolateral prefrontal cortex impairs antisaccade performance. Experimental brain research Experimentelle Hirnforschung Expérimentation cérébrale. 2008 10;190(4):463–73. Available from: http://www.ncbi.nlm.nih.gov/pubmed/18641976. 18641976 10.1007/s00221-008-1488-4

[57] Funahashi S, Chafee MV, Goldman-Rakic PS. Prefrontal neuronal activity in rhesus monkeys performing a delayed anti-saccade task. Nature. 1993 11;365:753–756. Available from: http://www.ncbi.nlm.nih.gov/pubmed/8413653. 10.1038/365753a0 8413653

[58] Johnston K, Everling S. Monkey dorsolateral prefrontal cortex sends task-selective signals directly to the superior colliculus. The Journal of neuroscience: the official journal of the Society for Neuroscience. 2006 11;26(48):12471–8. Available from: http://www.ncbi.nlm.nih.gov/pubmed/17135409. 10.1523/JNEUROSCI.4101-06.2006 17135409 PMC6674911

[59] Munoz DP, Everling S. Look away: the anti-saccade task and the voluntary control of eye movement. Nature Reviews Neuroscience. 2004 3;5(3):218–228. Available from: http://www.ncbi.nlm.nih.gov/pubmed/14976521. 10.1038/nrn1345 14976521

[60] Schlag-Rey M Amador N Sanchez H Schlag J. Antisaccade performance predicted by neuronal activity in the supplementary eye field. Nature.1997 12;390:398 Available from: http://www.ncbi.nlm.nih.gov/pubmed/9389478. 10.1038/37114 9389478

[61] Congdon E, Constable RT, Lesch KP, Canli T. Influence of SLC6A3 and COMT variation on neural activation during response inhibition. Biological Psychology. 2009 7;81(3):144–152. Available from: 10.1016/j.biopsycho.2009.03.005. 10.1016/j.biopsycho.2009.03.005 19482231 PMC2689843

[62] Aron AR, Behrens TE, Smith S, Frank MJ, Poldrack RA. Triangulating a cognitive control network using diffusion-weighted magnetic resonance imaging (MRI) and functional MRI. The Journal of neuroscience: the official journal of the Society for Neuroscience. 2007 04;27(14):3743–3752. Available from: http://www.ncbi.nlm.nih.gov/pubmed/17409238. 10.1523/JNEUROSCI.0519-07.2007 17409238 PMC6672420

[63] Isoda M, Hikosaka O. Switching from automatic to controlled action by monkey medial frontal cortex. Nature neuroscience. 2007 01;10(2):240–248. Available from: http://www.ncbi.nlm.nih.gov/pubmed/17237780. 10.1038/nn1830 17237780

[64] Egner T, Ely S, Grinband J. Going, going, gone: characterizing the time-course of congruency sequence effects. Frontiers in psychology. 2010 1;1:154 Available from: http://journal.frontiersin.org/article/10.3389/fpsyg.2010.00154/abstract. 21833220 10.3389/fpsyg.2010.00154 PMC3153769

[65] King JA, Korb FM, von Cramon DY, Ullsperger M. Post-error behavioral adjustments are facilitated by activation and suppression of task-relevant and task-irrelevant information processing. The Journal of neuroscience: the official journal of the Society for Neuroscience. 2010 9;30(38):12759–69. Available from: http://www.jneurosci.org/content/30/38/12759.short. 10.1523/JNEUROSCI.3274-10.2010 20861380 PMC6633589

[66] Bissett PG, Logan GD. Post-stop-signal slowing: strategies dominate reflexes and implicit learning. Journal of experimental psychology Human perception and performance. 2012 6;38(3):746–57. Available from: http://www.ncbi.nlm.nih.gov/pubmed/21895385. 10.1037/a0025429 21895385

[67] Cavanagh JF, Wiecki TV, Kochar A, Frank MJ. Eye Tracking and Pupillometry Are Indicators of Dissociable Latent Decision Processes. Journal of experimental psychology General. 2014 3;Available from: http://www.ncbi.nlm.nih.gov/pubmed/24548281. 10.1037/a0035813 PMC4114997 24548281

