# Supplementary material for: A Computational Cognitive Biomarker for Early-Stage Huntington’s Disease
Source: PLoS One. 2016 Feb 12;11(2):e0148409. doi: 10.1371/journal.pone.0148409 (PMC4752511; doi:10.1371/journal.pone.0148409)
Supplement: S1 File — Algorithm A, Algorithm to draw a single antisaccade trial. InvGaussian(μ, λ) is the first-passage-time distribution for a Wiener diffusion process with drift μ and a single upper threshold λ. t is a constant corresponding to non-decision time. texec is a second constant that captures the time needed to implement additional processing on antisaccade versus prosaccade trials. By running this algorithm 10000 times for each parameter setting and using kernel density estimation on the simulated RTs, we approximated a likelihood function. Prosaccade trials were fit using only the prepotent accumulator for which a closed-form solution is available. The parameters a, t and vpre are thus constrained by prosaccade as well as antisaccade trials while the parameters vstop, vexec, and texec are only constrained by the latter. Figure A, Bar-plots of behavioral mean reaction time in seconds across different groups in the antisaccade condition. Ellipses in grey represent mean RT of trials simulated from models fit to each individual subject. Height of the ellipses represents standard-deviation. Figure B, Bar-plot comparing Area under the ROC Curve (AUC) of a logistic regression classifier trained on different data to predict HC and pre-HD. Error-bars represent standard deviation. Figure C, Bar-plot comparing Area under the ROC Curve (AUC) of a logistic regression classifier trained on different data to predict pre-HD-A and pre-HD-B. Error-bars represent standard deviation. Table A, AIC values of different model configurations. Lower AIC values represent a better trade-off between parsimony and model fit. Table B, Multiple Comparison of Means of t parameter—Tukey HSD. Table C, Multiple Comparison of Means of vpro parameter—Tukey HSD. Table D, Multiple Comparison of Means of vstop parameter—Tukey HSD. Table E, Multiple Comparison of Means of vexec parameter—Tukey HSD. Table F, Multiple Comparison of Means of texec parameter—Tukey HSD. Table G, Multiple Comparison of Means of amean pa [file pone.0148409.s001.pdf]

A computational cognitive biomarker for early-stage Huntington's disease

Supporting Information

Model details

Prosaccade trials were fit with an inverse Gaussian distribution. For antisaccade trials, however, a closed-form solution is intractable so we used simulation to estimate a likelihood function. The algorithm to simulate a single antisaccade trial can be seen in Algorithm A.

---

function SAMPLEANTISACCADETRIAL(v_{pre}, v_{inhib}, v_{exec}, a, t, t_{exec})
  $RT_{pre} \sim InvGauss(v_{pre}, a) + t$
  $RT_{inhib} \sim InvGauss(v_{inhib}, a) + t$
  $RT_{exec} \sim InvGauss(v_{exec}, a) + t + t_{exec}$
  if $RT_{inhib} \leq RTpre$ **then**
    $RT_{pre} \leftarrow inf$
  if $RT_{pre} \leq RT_{exec}$ **then**
    $error \leftarrow true$
    $RT \leftarrow RT_{pre}$
  else
    $error \leftarrow false$
    $RT \leftarrow RT_{exec}$
  return $RT, error$

Algorithm A

Algorithm to draw a single antisaccade trial. InvGaussian(μ, λ) is the first-passage-time distribution for a Wiener diffusion process with drift μ and a single upper threshold λ. t is a constant corresponding to non-decision time. t_{exec} is a second constant that captures the time needed to implement additional processing on antisaccade versus prosaccade trials. By running this algorithm 10000 times for each parameter setting and using kernel density estimation on the simulated RTs, we approximated a likelihood function. Prosaccade trials were fit using only the prepotent accumulator for which a closed-form solution is available. The parameters a, t and v_{pre} are thus constrained by prosaccade as well as antisaccade trials while the parameters v_{stop}, v_{exec}, and t_{exec} are only constrained by the latter.

Priors were placed on each parameter to constrain the parameter space to sensible values (i.e. all parameters have positive support) as well to apply mild regularization. The results obtained in the analysis do not rely on the specific choice of priors.

$$t \sim \Gamma(0.3, 1.)$$
$$a \sim \Gamma(2.0, 0.75)$$
$$v_{pro} \sim \Gamma(0.5, 1)$$
$$v_{stop} \sim \Gamma(1.5, 1)$$
$$v_{anti} \sim \Gamma(0.5, 1)$$
$$t_{anti} \sim \Gamma(0.1, 1)$$

Usually, a Gamma distribution (Γ in the table above) is parameterized by a shape and a rate parameter. Here we use an alternative paratemerization that uses location (i.e. the mean of the distribution) and scale (i.e. the standard deviation of the distribution) instead. To get the shape and rate parameters from location and scale the following transformation can be applied [5]:

$$shape = \frac{loc^2}{scale^2}$$
$$rate = \frac{loc}{scale^2}$$

Model fit

To further test whether the model could reproduce the basic group patterns observed we simulated RTs from the model using parameters fit to each individual subject. These simulated subject RTs were then compared to the mean RTs in antisaccade trials observed behaviorally (Fig A). As can be seen, mean RT is captured well across the three groups and statistical tests reveal the same pattern as observed on behavioral data (not shown).

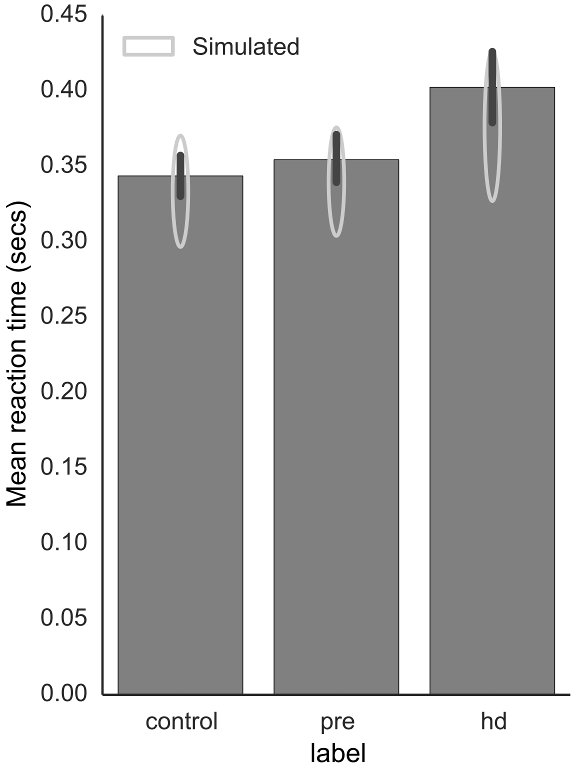

Figure A

Bar-plots of behavioral mean reaction time in seconds across different groups in the antisaccade condition. Ellipses in grey represent mean RT of trials simulated from models fit to each individual subject. Height of the ellipses represents standard-deviation.

As various different model formulations appear feasible we performed model comparison using AIC [4] to determine the most parsimonious model that still produces good fit. As can be seen in Table A, a model that has a specific stop-process that suppresses the prepotent prosaccade response in antisaccade trials produces better (lower) AIC values. While a single threshold is slightly favored for this model, we report values for the model that allows separate thresholds for pro and antisaccade trials based on previous research [2, 3]. Results reported above hold in both model configurations.

Model type	AIC
No stop process, single threshold	-69.49
No stop process, separate thresholds	-76.13
Stop process, single threshold	-83.63
Stop process, separate thresholds	-82.96

Table A

AIC values of different model configurations. Lower AIC values represent a better trade-off between parsimony and model fit.

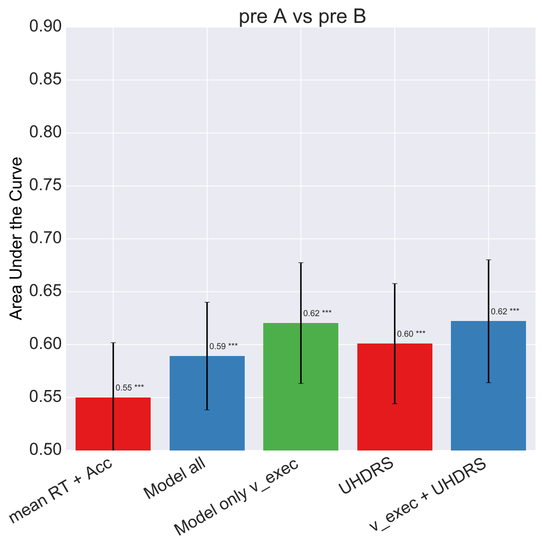

Machine Learning results

Figure B and Figure C contain additional classifier results.

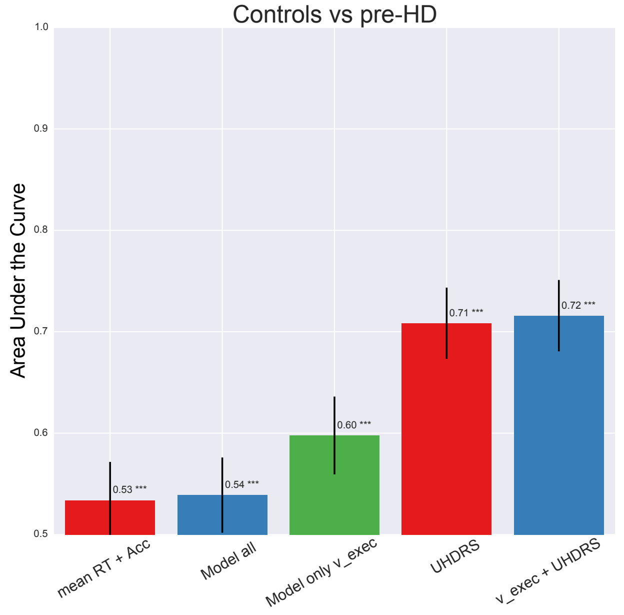

Figure B

Bar-plot comparing Area under the ROC Curve (AUC) of a logistic regression classifier trained on different data to predict HC and pre-HD. Error-bars represent standard deviation.

Figure C

Bar-plot comparing Area under the ROC Curve (AUC) of a logistic regression classifier trained on different data to predict pre-HD-A and pre-HD-B. Error-bars represent standard deviation.

Comparisons of all parameters

Below we show a more thorough examination of how all parameters differ between groups. To reduce multiple comparison errors, all results are Tukey HSD corrected. Specifically, comparisons of model parameter t can be found in table Table B, v_{pro} in Table C, v_{exec} in Table D, v_{stop} in Table E, t_{exec} in Table F, a_{mean} in Table G, and a_{diff} in Table H.

group1	group2	meandiff	lower	upper	reject
controls	pre	-0.0059	-0.0238	0.012	False
controls	HD	-0.0245	-0.0423	-0.0066	True
pre	HD	-0.0186	-0.0364	-0.0007	True

Table B

Multiple Comparison of Means of t parameter - Tukey HSD

group1	group2	meandiff	lower	upper	reject
controls	pre	-0.1419	-0.4908	0.207	False
controls	HD	-0.9215	-1.2683	-0.5748	True
pre	HD	-0.7796	-1.1271	-0.4321	True

Table C

Multiple Comparison of Means of v_{pro} parameter - Tukey HSD

group1	group2	meandiff	lower	upper	reject
controls	pre	-0.0106	-0.7374	0.7163	False
controls	HD	-1.798	-2.5205	-1.0756	True
pre	HD	-1.7875	-2.5114	-1.0635	True

Table D

Multiple Comparison of Means of v_{stop} parameter - Tukey HSD

group1	group2	meandiff	lower	upper	reject
controls	pre	-0.8827	-1.6259	-0.1395	True
controls	HD	-4.0628	-4.8015	-3.3241	True
pre	HD	-3.1801	-3.9203	-2.4399	True

Table E

Multiple Comparison of Means of v_{exec} parameter - Tukey HSD

group1	group2	meandiff	lower	upper	reject
controls	pre	0.0003	-0.0299	0.0306	False
controls	HD	0.0345	0.0044	0.0646	True
pre	HD	0.0342	0.004	0.0643	True

Table F

Multiple Comparison of Means of t_{exec} parameter - Tukey HSD

group1	group2	meandiff	lower	upper	reject
controls	pre	-0.0519	-0.2747	0.171	False
controls	HD	-0.341	-0.5625	-0.1194	True
pre	HD	-0.2891	-0.5111	-0.0671	True

Table G

Multiple Comparison of Means of a_{mean} parameter - Tukey HSD

group1	group2	meandiff	lower	upper	reject
controls	pre	-0.0366	-0.1	0.0267	False
controls	HD	-0.118	-0.181	-0.055	True
pre	HD	-0.0814	-0.1445	-0.0183	True

Table H

Multiple Comparison of Means of a_{diff} parameter - Tukey HSD

References

1. Ratcliff R, McKoon G. The diffusion decision model: theory and data for two-choice decision tasks. Neural computation. 2008 Apr;20:873–922. Available from: http://www.ncbi.nlm.nih.gov/pubmed/18085991.
2. Wiecki TV, Frank MJ. A computational model of inhibitory control in frontal cortex and basal ganglia. Psychological Review. 2013 Apr;120(2):329–355. Available from: http://www.ncbi.nlm.nih.gov/pubmed/23586447.
3. Cavanagh JF, Wiecki TV, Cohen MX, Figueroa CM, Samanta J, Sherman SJ, et al. Subthalamic nucleus stimulation reverses mediofrontal influence over decision threshold. Nature neuroscience. 2011 Sep;14:1462–1467. Available from: http://www.ncbi.nlm.nih.gov/pubmed/21946325.
4. Schwarz G. Estimating the Dimension of a Model. The Annals of Statistics. 1978 Mar;6(2):461–464. Available from: http://projecteuclid.org/euclid.aos/1176344136.

5. Kruschke J. Doing Bayesian data analysis: A tutorial introduction with R and BUGS. Academic Press / Elsevier; 2010. Available from: http://books.google.com/books?hl=en&lr=&id=ZRMJ-CebFm4C&oi=fnd&pg=PP2&dq=kruschke+doing+bayesian+data+analysis&ots=DsCCPI6uAW&sig=05YmmOLJ8DbRLwvtXxwWyIg0Eq0.
